# Beliefs and Attitudes Towards Sunscreen and Sun Protection Practices Among South Asians in British Columbia: A Survey Study

**DOI:** 10.1111/phpp.70029

**Published:** 2025-06-11

**Authors:** Harman Toor, Mahtab S. Gill, Tashmeeta Ahad

**Affiliations:** ^1^ Department of Dermatology and Skin Science University of British Columbia Vancouver British Columbia Canada; ^2^ Faculty of Medicine University of British Columbia Vancouver British Columbia Canada; ^3^ Vancouver Coastal Health Research Institute Vancouver British Columbia Canada

**Keywords:** Asian people, attitudes, Canada, health knowledge, practice, public health, sunscreening agents

## Abstract

**Background/Purpose:**

South Asians form a substantial proportion of Canada's Skin of Color (SOC) population, yet limited knowledge exists about their sun protection beliefs, attitudes, and practices. This study aimed to explore these factors.

**Methods:**

A cross‐sectional survey was conducted from April to October 2024 to evaluate sun protection factors among South Asian adults in British Columbia. Sixty‐two participants were recruited, with a 90% response rate, using convenience sampling through outreach events and social media.

**Results:**

Most were born in Canada or South Asia, and 41% were aged 25–34 years. More females (43%) used sunscreen daily compared to males (12%). Although 69% believed in sun protection health benefits, 30% rarely or never wore sunscreen. Motivators included preventing sunburn (68%), maintaining youthful skin (65%), and reducing skin cancer risk (63%). A minority (25%) used sun protection to achieve a lighter skin complexion. Most were unsure if religious coverings provided adequate protection. Social media (41%) was the most common source of information. Barriers included forgetfulness (69%), low perceived risk (41%), discomfort (24%), and cosmetic concerns (17%) like “white cast” (61%). Preferences for organic versus inorganic sunscreens were mixed.

**Conclusion:**

Most valued sun protection for health, preventing sunburn, photoaging, and reducing skin cancer risk, despite lower baseline risk relative to those with European ancestry. Social media presents an opportunity for targeted outreach. Australia has recently tailored sunscreen recommendations based on skin type, recognizing variability in risks and sun protective needs among their diverse population. Similar approaches could be considered in Canada to better address the needs of SOC populations.

## 1 Background

Individuals with South Asian ancestry represent a significant proportion of Canada's Skin of Color population. South Asians represent the largest new immigrant population to Canada and have a long history of migration to British Columbia, spanning over a century [1]. In addition, within dermatology, there has been an increased emphasis on inclusivity and representation of skin of color groups.

The Canadian Dermatology Association recommends sun protection for all skin types [2]; however, the degree of protective benefits varies based on genetic factors related to ancestry and skin tone. Notably, Australia has recently tailored sunscreen recommendations based on skin type, recognizing variability in risks, and sun protective needs among their diverse population [3].

Despite a large population, there is limited knowledge about the sun protection beliefs, attitudes, and behaviors among South Asians in Canada. A deeper understanding of these factors may help inform more targeted public health initiatives. To explore these factors, we conducted a survey study among a sample of South Asians residing in British Columbia.

## 2 Methods

A cross‐sectional questionnaire survey was used to explore the beliefs, attitudes, behaviors, and knowledge regarding sunscreens and sun protection among a sample of South Asians residing in British Columbia. This study received ethics approval from the University of British Columbia Behavioural Research Ethics Board (study number H23‐02920). All participants were provided with an informed consent form prior to participation, and participation was voluntary and results confidential. Participants were also able to withdraw from the study at any point throughout the questionnaire.

The survey collected data from April to October 2024. It recorded 62 participants, using convenience sampling, 18 years and older living in British Columbia. The survey response rate was 90%, with 56 participants completing the entire survey. Eligible participants were individuals who self‐identified as South Asian and resided in British Columbia at the time of the study. Participants were recruited via community outreach events through the UBC Medical Student South Asian Health Club. Additional participants were recruited through social media platforms. The survey was completed via an online form at events in Vancouver, Surrey, and Prince George, British Columbia. The survey consisted of 38 questions, including multiple‐choice, modified Likert‐scale, and short answer.

## 3 Results

### 3.1 Demographics

Of the survey participants, 53% were born in Canada and 44% were born in South Asia. The majority (41%) were 25–34 years of age, although 23% of participants were older than 45 years. 59% were female and 41% were male. More than half (56%) of participants spoke a language other than English at home, with most of those being Punjabi speakers. 77% had either lived in British Columbia all their life or had been in the province for more than 20 years.

Most participants had a Bachelor's degree and most were employed full time. Only 8% reported having an occupation that involved being outdoors, with exposure to the sun all day.

With respect to skin type and tone, the majority, 54% described their skin tone as medium, 43% as fair, and 4% as very fair. These categories were used to facilitate ease of completion for participants. The Fitzpatrick classification, which assesses skin based on response to sun exposure, was not used since it does not accurately reflect inherent skin tone [4]. See Table 1 for full demographic details.

**TABLE 1:** Demographics.

Country of birth	Percent	Count
Canada	53	27
South Asia	44	33
Other	3	2
Gender		
Male	41	25
Female	59	36
Age		
18–24	16	10
25–34	41	25
35–44	20	12
45–54	10	6
55–64	5	3
65+	8	5
Primary language		
English	44	27
Punjabi	48	29
Hindi	3	2
Gujrati	2	1
Bengali	3	2
Years living in British Columbia		
All of life	49	30
1 to 5 years	7	4
6 to 10 years	12	7
11 to 20 years	5	3
20+ years	28	17
Educational attainment		
High school or below	17	10
College or vocational	10	6
Bachelors	43	26
Masters	15	9
Doctorate	15	9
Outdoor occupation		
All day	8	3
Part of the day	21	8
Rarely	49	19
Never	23	9
Occupational status		
Employed full time	62	38
Employed part time	2	1
Student	26	16
Unemployed	3	2
Retired	7	4
Skin type/Tone		
Very fair	4	2
Fair	43	24
Medium	54	30
Dark	0	0

### 3.2 Behaviors

#### 3.2.1 Frequency of Sunscreen Use

Among participants, 30% reported rarely or never wearing sunscreen, while 22% used it occasionally and 48% reported using sunscreen often or daily (Table 2). Females were more likely to report using sunscreen daily (43%) compared to males (12%).

**TABLE 2:** Sunscreen usage behaviors.

Question	Percent	Count
How often do you use sunscreen?		
Never	13	8
Rarely	17	10
Occasionally	22	13
Often	18	11
Daily	30	18
Is using sunscreen part of your daily routine (i.e., like brushing teeth)?		
Yes	41	23
No	59	33
If you have children under 18, do you take specific sun protection measures for them?		
Yes	18	10
No	5	3
Not applicable	77	43
How often do you wear sun protective clothing (e.g., long sleeves, wide‐brimmed hats) when exposed to the sun?		
Never	9	5
Rarely	25	14
Occasionally	34	19
Often	25	14
Daily	7	4
How often do you seek shade when outdoors during peak sun hours (10 AM to 4 PM)?		
Never	2	1
Rarely	7	4
Occasionally	38	21
Often	48	27
Daily	5	3

#### 3.2.2 Usage Behaviors Across Different Age Groups

75% of elder participants (65 years and older) reported never using sunscreen, whereas in contrast, 77% of parents took specific sun protection measures for their children under 18.

#### 3.2.3 Alternative Sun Protection Behaviors

With respect to other sun protective behaviors, 25% of respondents reported often wearing sun protective clothing, such as long sleeves and hats, while 7% reported always using such clothing when exposed to the sun. Additionally, most respondents indicated they seek shade during peak sun hours, with 38% doing so occasionally, 48% often, and 5% daily (Table 2).

#### 3.2.4 Self‐Reported Frequency of Sunburning

With respect to sunburn, most respondents reported experiencing it rarely (34%) or never (32%). A smaller proportion reported sunburn once a year or less (14%), 2 or more times per year (18%), or always (2%) when exposed to the sun.

### 3.3 Beliefs and Attitudes

Most participants (69%) viewed sun protection as beneficial to overall health. 65% engaged in sun protection to help maintain youthful skin, and 68% reported engaging in sun protection to prevent sun burn. In addition, 63% believed their engagement in sun protection would reduce their risk for skin cancer (Table 3).

**TABLE 3:** Attitudes around sunscreen usage and sun protection.

Question	Percent	Count
Please rate your agreement with the statement “Sun protection is important for my overall health.”		
Strongly disagree	8	5
Disagree	8	5
Neutral	15	9
Agree	42	25
Strongly agree	27	16
Please rate your agreement with the statement: “I engage in sun protective behaviors to help maintain youthful looking skin.”		
Strongly disagree	9	5
Disagree	13	7
Neutral	14	8
Agree	38	21
Strongly agree	27	15
Please rate your agreement with the statement: “I engage in sun protection practices to prevent sunburn.”		
Strongly disagree	4	2
Disagree	7	4
Neutral	21	12
Agree	48	27
Strongly agree	20	11
Please rate your agreement with the statement “I engage in sun protection practices to prevent skin cancer.”		
Strongly disagree	9	1
Disagree	9	22
Neutral	20	16
Agree	43	15
Strongly agree	20	2
Have you ever experienced sunburn? If so, how frequently?		
Never	32	18
Rarely (once every few years)	34	19
Occasionally (once a year)	14	8
Often (2 or more times per year)	18	10
Always (every time I am exposed to the sun)	2	1
What are the primary reasons you might not use sunscreen (select all that apply)		
Forgetfulness	69	20
I perceive a low risk of sun damage	41	12
They feel uncomfortable	24	7
I have never considered using them	21	6
Do not like the appearance of sun protection products on the skin	17	5
Concerns about the presence of harmful chemicals	14	4
I doubt their effectiveness	10	3
Too expensive	7	2
Other	7	2
Limited access to sun protective measures	0	0
Do you have preferences for specific types of sunscreen (e.g., mineral/inorganic, chemical/organic, water‐resistant)?		
Yes	34	19
No	34	19
Don't know	32	18
Have you experienced a white cast on your skin (whitish film) with sunscreen?		
Yes	61	34
No	29	16
Don't know	11	6
Are you concerned about chemicals in sunscreens, causing other health problems?		
Yes	36	20
No	64	36
Do you believe that head coverings (e.g., turbans, head scarf or hijab) provide adequate protection against the sun?		
Yes	30	17
No	30	17
Not sure	39	22

#### 3.3.1 Reasons for Not Using Sunscreen

The most common reasons for not using sunscreen selected by participants included forgetfulness (69%), a low perceived risk of sun damage (41%) and that they felt uncomfortable (24%) or did not like the appearance of the product (17%) on their skin.

#### 3.3.2 Sunscreen Preferences and User Experience

Although there was no trend toward a preference for inorganic (“mineral”) or organic (“chemical”) sunscreens, among those surveyed, 61% had at one point experienced a “white cast” when using a sunscreen.

#### 3.3.3 Concerns Regarding Sunscreen Use

Just over a third of participants (36%) had concerns regarding chemicals in sunscreen products potentially causing other health problems. Only 10% doubted the effectiveness of sunscreens.

#### 3.3.4 Perceptions of Sun Protection and Faith‐Based Head Coverings

Given that many South Asians follow faith practices that involve wearing head coverings, such as turbans or headscarves, there was notable uncertainty regarding their effectiveness in providing sun protection. A majority (39%) were unsure whether these coverings provided adequate protection from the sun, while 30% believed they did, and another 30% believed they did not.

#### 3.3.5 Colorism

Colorism is prevalent in some South Asian communities and is often reflected in media and advertisements, where lighter skin is portrayed as more desirable, particularly in Bollywood [5]. In this survey, most respondents disagreed (32%) or strongly disagreed (18%) with using sun protection to achieve lighter skin (Table 4). However, a minority (25%) did report using sun protection to achieve or maintain a lighter complexion, potentially indicating the ongoing influence of colorism.

**TABLE 4:** Sunscreen usage and colorism.

Question	Percent	Count
Please rate your agreement with the statement “I engage in sun protection practices to maintain or achieve lighter skin.”		
Strongly disagree	18	10
Disagree	32	18
Neutral	25	14
Agree	21	12
Strongly agree	4	2

### 3.4 Knowledge and Special Factors

57% reported awareness of the UV index and its significance; however, most reported never (57%) or rarely (11%) checking it (Table 5). Despite this, the majority (66%) did report weather (i.e., cloudy vs. sunny days) and season affected their use of sunscreens.

**TABLE 5:** Knowledge and special factors.

Question	Percent	Count
Are you aware of the UV index and its significance?		
Yes	57	32
No	43	24
How often do you check the UV index before going outdoors?		
Never	57	32
Rarely	11	6
Occasionally	29	16
Often	4	2
Does the weather (e.g., cloudy vs. sunny days) or season affect your sun protection practices?		
Yes	66	37
No	34	19
Do you have a medical or dermatologic condition that makes engaging in sun protection particularly important?		
Yes	14	8
No	86	48

The majority (86%) reported not having a medical or dermatologic condition that made engaging in sun protection particularly important. Of those that did (14%), they reported pigmentary concerns like hyperpigmentation and melasma, as well as photosensitive disorders like lupus. Two reported eczema and psoriasis, respectively, and one reported cold sores.

### 3.5 Information Sources and Future Education

Social media was the most common source of sunscreen information (41%), followed by family or friends (34%) and traditional media (30%) (Table 6). A smaller proportion received information from dermatologists (25%) and family physicians (21%). Notably, 21% had not received any information on sun protection. Although only 29% engaged with sun protection content on social media, 66% expressed interest in learning more through educational programs or outreach initiatives.

**TABLE 6:** Information sources and future information.

Question	Percent	Count
Where do you typically receive information about sun protection practices? (Select all that apply)		
Social media (TikTok, Facebook, Instagram, etc.)	41	23
Family or friends	34	19
Traditional media (TV, magazines, radio)	30	17
Dermatologist	25	14
I have not received information on sun protection practices before	21	12
Family doctor or primary care provider	21	12
Pharmacist	7	4
Nurses	5	3
Other (please specify)	5	3
Community organizations	4	2
Do you follow or engage with social media content related to sun protection?		
Yes	29	16
No	71	40
Would you be interested in educational programs about sun protection?		
Yes	66	37
No	34	19

## 4 Discussion

In this study of South Asians, females and those of younger age (18–34) were more likely to report frequent or daily sunscreen use. The majority of participants reported experiencing a white cast when using sunscreen, although results were mixed with regard to a clear preference for mineral or organic formulations. This highlights a potential area for education, as organic sunscreens are generally less likely to cause a white cast [6]. Our study did not distinguish whether the sunscreen used by participants was part of a makeup product or a standalone sunscreen, nor did it investigate the specific filters in the products. Exploring these aspects could be valuable in future research, particularly given the growing understanding of the role of the visible light spectrum in skin of color patients.

The majority of participants (69%) believed that sun protection was important for overall health and for reducing the risk of skin cancer, despite the fact that UV‐related skin cancers are far less common in otherwise healthy individuals with skin of color [7]. This perceived risk may stem from the lack of tailored public health messaging that considers variations in skin type. However, there may be other compelling reasons for sun protection in those with skin of color, including preventing dyspigmentation, mitigation of skin photoaging, and part of the management of photosensitive skin conditions [8]. Notably, recent Australian guidelines have acknowledged that a one‐size‐fits‐all approach to sun protection may not be appropriate [3]. They now recommend against routine photoprotection in individuals with darker skin types, citing low risk of UV‐related skin cancer but relatively higher risks for vitamin D deficiency [3]. In our study, 63% of participants reported engaging in sun protection practices to prevent skin cancer, despite having a very low baseline risk. This may reflect the influence of generalized sun safety messaging in Canada, which historically has been aimed at individuals of European ancestry. Future educational campaigns could explain the different reasons why sunscreen and sun protection are useful in a more individualized approach, paralleling Australian guidelines, considering factors such as diversity of skin type and ancestry. For example, for richly pigmented non‐European ancestry patients, messaging could be tailored to the benefits of sun protection to prevent dyspigmentation rather than helping them prevent UV‐related skin cancers.

There was uncertainty (39%) regarding whether faith‐based head coverings could provide adequate sun protection amongst our participants. However, the protection provided by a turban or headscarf may not be comprehensive. While it can shield the scalp and parts of the forehead, it may not adequately protect other areas of the face, such as the nose, cheeks, and neck. The general recommendation for wide‐brimmed hats may not be feasible for these groups.

The most common source of information was social media and family or friends. Although social media is an accessible source of information, the reliability of the information provided is highly variable [9]. This underscores the importance of the social media presence of health care professionals and public health organizations to provide reliable and valid information to the public. This is an opportunity for dermatology societies and organizations to further leverage educational initiatives.

### 4.1 Limitations

This study has several limitations. The sample size is small, and participants were recruited through convenience sampling, which may limit the generalizability of the findings to the broader South Asian and skin of color population in Canada. Self‐reported data are subject to recall and social desirability biases, potentially affecting the accuracy of reported behaviors and beliefs. Additionally, while the survey questions were designed to assess key variables, they may not have fully captured all factors influencing sunscreen and sun protection behaviors.

## 5 Conclusion

This cross‐sectional survey provides insights into sunscreen and sun protection beliefs, attitudes, behaviors, and knowledge among South Asians in British Columbia. Many participants reported the importance of sun protection for overall health, preventing skin photoaging, sun burn, and preventing skin cancer even though their baseline risk is lower relative to European ancestry populations. Despite these beliefs, a significant proportion reported limited sunscreen use, influenced by forgetfulness, discomfort, lack of perceived benefit, and cosmesis with concerns like the “white cast.”

Given the diverse populations in Canada, future public health efforts regarding sun safety could consider tailored recommendations based on skin type and ancestry to enhance inclusivity and accuracy of sun safety messaging.

## Conflicts of Interest

The authors declare no conflicts of interest.

## Data Availability

The data that support the findings of this study are available from the corresponding author upon reasonable request.
